# Interaction of Stellate Cells with Pancreatic Carcinoma Cells

**DOI:** 10.3390/cancers2031661

**Published:** 2010-09-09

**Authors:** Hansjörg Habisch, Shaoxia Zhou, Marco Siech, Max G. Bachem

**Affiliations:** 1 Department Clinical Chemistry and Central Laboratory, University of Ulm, Germany; E-Mails: hansjoerg.habisch@uniklinik-ulm.de (H.H); shaoxia.zhou@uniklinik-ulm.de (S.Z.); 2 Department of surgery, Ostalbklinikum Aalen, Germany; E-Mail: marco.siech@ostalb-klinikum.de (M.S.)

**Keywords:** pancreas carcinoma, pancreatic stellate cell, tumor desmoplasia, EMMPRIN, chemoresistance

## Abstract

Pancreatic cancer is characterized by its late detection, aggressive growth, intense infiltration into adjacent tissue, early metastasis, resistance to chemo- and radiotherapy and a strong “desmoplastic reaction”. The dense stroma surrounding carcinoma cells is composed of fibroblasts, activated stellate cells (myofibroblast-like cells), various inflammatory cells, proliferating vascular structures, collagens and fibronectin. In particular the cellular components of the stroma produce the tumor microenvironment, which plays a critical role in tumor growth, invasion, spreading, metastasis, angiogenesis, inhibition of anoikis, and chemoresistance. Fibroblasts, myofibroblasts and activated stellate cells produce the extracellular matrix components and are thought to interact actively with tumor cells, thereby promoting cancer progression. In this review, we discuss our current understanding of the role of pancreatic stellate cells (PSC) in the desmoplastic response of pancreas cancer and the effects of PSC on tumor progression, metastasis and drug resistance. Finally we present some novel ideas for tumor therapy by interfering with the cancer cell-host interaction.

## 1. Introduction

In the United States of America, Europe and Japan, the incidence of pancreatic cancer has risen slowly during the last few decades. Pancreatic ductal adenocarcinoma (PDA) is now the fourth leading cause of cancer related death among both men and women in the U.S. [1]. Because this cancer shows no symptoms in its early stage and has therefore a low probability of diagnosis, approximately 80–90% of the patients present with local infiltration or metastatic disease at the time of initial diagnosis [2]. Therefore only 15–20% of the patients are candidates for surgical resection, which is the only chance for cure. The median survival time of patients with metastatic pancreas cancer is <6 months and the overall five-year survival rate is less than 5%. 

More than 90% of the pancreas cancers represent PDA, which are characterized by a late detection, an aggressive growth, local invasion into adjacent tissue, a rapid progression, early systemic dissemination [3], late diagnosis and a relative resistance to conventional chemo- and radiotherapy [4,5]. After surgical resection local recurrence occurs in the majority of patients. In addition, a strong “desmoplastic reaction” is characteristic for PDA [6,7]. Fibroblasts, myofibroblasts and activated stellate cells produce the different connective tissue components such as collagens, fibronectin and proteoglycans [8]. As shown by Immamura *et al.* [8] in pancreas cancer and tumor associated chronic pancreatitis, the collagen content is 3-fold higher compared to normal pancreas. In addition, the proportion of the collagen types I, III and V is comparable to ethanol induced chronic pancreatitis, tumor associated chronic pancreatitis and pancreas cancer [8]. Whereas in pancreas cancer, collagen synthesis is associated with spindle shaped cells (fibroblasts and myofibroblasts), matrix-metalloproteinases (MMPs) and tissue-inhibitors of MMPs (TIMPs) are produced by both, stromal and tumor cells [9]. 

In his “Frank Brooks Memorial State of the Art Lecture in basic Sciences” at the 2001 Annual APA-meeting, M.G. Bachem presented data for the first time indicating an interaction of PSC with tumor cells. One year later, Yen *et al*. [10] described a pronounced increase in the number of α-smooth muscle actin positive cells in PDA and suggested that these cells might represent activated PSC producing the connective tissue surrounding carcinoma cells. Thereafter several research groups studied the role of PSC in pancreas cancer [11,12,13,14]. 

## 2. Pancreatic Stellate Cells

**Figure 1:**
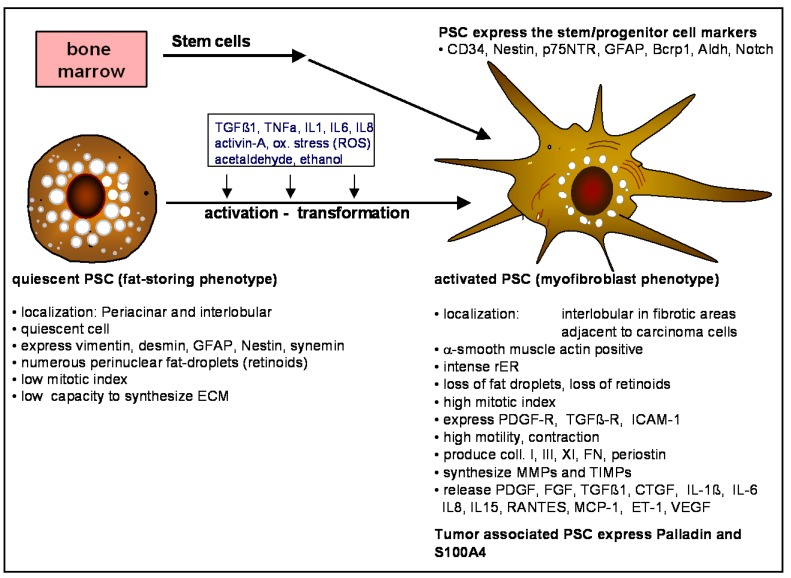
Characteristics of quiescent (fat-storing phenotype) and activated PSC (myofibroblast-like phenotype). In acute and chronic pancreatitis, but also in pancreas carcinomas, PSC change their phenotype from a quiescent fat-storing phenotype to a highly active myofibroblast-like phenotype. Hereby the cells lose their Vitamin A containing fat droplets, express other cytofilaments, increase their proliferation rate and produce growth factors, cytokines, connective tissue, MMPs and TIMPs. In addition, as we have learned recently from animal models, part of the activated PSC originate from stem/progenitor cells of bone marrow.

In an earlier report, fibroblast-like cells were suggested to be responsible for the collagen synthesis resulting in pancreas fibrosis [15]. However, as shown later, the matrix producing cells in pancreas express α-smooth muscle actin (αSMA) and show similarities to activated hepatic stellate cells (HSC) or myofibroblast-like cells [16,17]; for a review see [18]. Normal fibroblasts do not express desmin or αSMA. In addition, by using microarray technology to analyze the gene expression profile of (i) normal fibroblasts; (ii) activated HSC and (iii) activated PSC, respectively, significant differences between stellate cells (HSC and PSC) and fibroblasts could be demonstrated [19]. Vitamin A storing cells have been found in different organs of vertebrates such as the liver, pancreas, kidney, lung, skin and others. HSC have long been known to play a major role in repair after injury and in liver fibrogenesis [20,21]. The cellular vitamin A content, primarily retinyl palmitate, is visible during excitation with UV-light as a rapidly fading fluorescence. Some cytoskeleton proteins might also be used to identify these cells (see Figure 1). PSC are of mesangial origin but, as we have learned recently in injury and cancer, part of these cells also originate from bone marrow (see below).

Vitamin A storing cells in the pancreas were first described in the year 1982 by Watari *et al.* using electron and fluorescence microscopy of mice pancreas tissue after vitamin A loading [22]. A few years later, vitamin A storing cells were found in normal human and rat pancreas and in fibrotic human pancreas [23]. In 1998 we, and the Apte-Wilson-Group in Sydney, isolated and characterized vitamin A storing stellate-shaped cells from rat and human pancreas [16,17]. Because of their similarity to HSC we named the cells pancreatic stellate cells [17]. In normal pancreas low numbers of quiescent fat‑storing PSCs can be detected interlobular and in the periacinar space [16,17]. Comparable to the stellate cell-activating mechanisms in liver injury, also in acute and chronic pancreatitis and in pancreas cancer (but also in primary culture), the cells are activated and change their phenotype (Figure 1). The fat storing phenotype of PSC is quiescent (low mitotic index, low capacity to produce matrix and growth factos), has numerous perinuclear fat droplets containing retinyl-palmitate and expresses the cytofilaments vimentin, desmin, glial fibrillary acidic protein (GFAP), Nestin and synemin (Figure 1).

**Figure 2:**
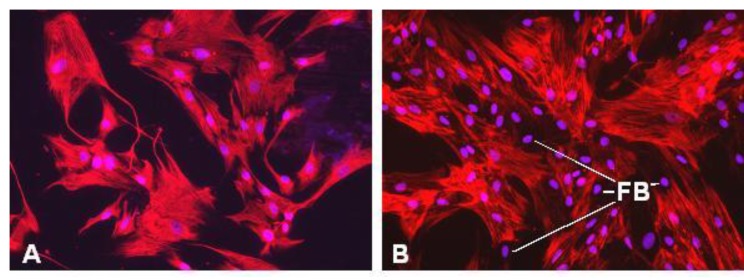
Immunofluorescence microscopy of cultured PSC, which have been isolated from pancreatic ductal adenocarcinoma. **(a)** vimentin immunofluorescence (red); **(b)** alpha‑smooth muscle actin immunofluorescence (red). Cell nuclei are stained blue (Hoechst 33258). FB, alpha-smooth muscle actin negative fibroblast.

In pancreas injury (e.g., acute and chronic pancreatitis), but also in pancreas carcinoma [24], the quiescent fat-storing phenotype of PSC loses its retinoids, develops a prominent rough endoplasmic reticulum and transforms into a highly active matrix producing myofibroblast-like cell (Figure 1). This cell type is primarily found in interlobular fibrotic areas or adjacent to carcinoma cells. The activated PSC (myofibroblast-like) are vimentin and αSMA positive, have a high mitotic index, express the receptors for PDGF and TGFß, express ICAM-I, are able to contract and move, produce the extracellular matrix components collagen I, III, XI, fibronectin and periostin, also synthesise MMPs and TIMPs and release the growth factors PDGF, FGF, TGFß1, CTGF, IL1ß, IL-6, IL-8, IL-15, Rantes, MCP1, ET1 and VEGF (see Figure 1). In addition, PSC which have been isolated from pancreas carcinomas also contain lipid droplets [14] and express vimentin and αSMA (Figure 2). These tumor derived PSCs also produce collagen I and III, fibronectin, growth factors, and proteases in significant amounts [13,14,24].

**Figure 3:**
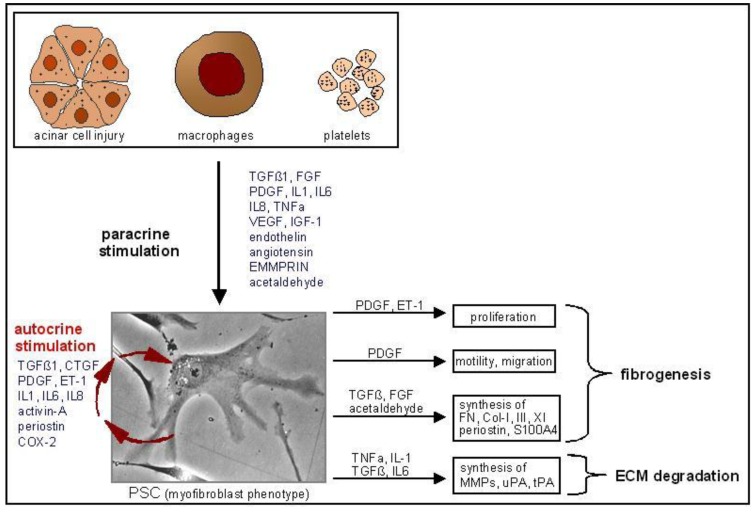
Paracrine and autocrine stimulation of activated pancreatic stellate cells in culture. Injured acinar cells, activated inflammatory cells, aggregating platelets, ethanol and acetaldehyde stimulate PSC proliferation, matrix- and MMP-synthesis and PSC motility. Via autocrine stimulatory loops PSC proliferation and ECM-synthesis are also stimulated. AM, adrenomedullin; SDF-1, stromal cell-derived factor-1; ET-1, endothelin-1.

Cell culture experiments have shown that TGFß, TNFα, IL-1, IL-6, IL-8, ethanol, acetaldehyde, and oxidative stress stimulate the transformation from the fat storing phenotype to the myofibroblast-like phenotype (Figure 1). Activated PSC are stimulated by injured acinar cells [25], aggregating platelets, inflammarory cells, ethanol and acetaldehyde to proliferate, produce matrix, and MMPs [26], and synthesize growth factors and cytokines (Figure 3) ([27]). The most important paracrine factors stimulating fibrogenesis in activated PSC are TGFß1, FGF, PDGF, ET-1, and acetaldehyde. TNFα, IL‑1, TGFß, and IL-6 are related to ECM degradation and remodeling (Figure 3). Because activated PSC synthesize TGFß1, CTGF, PDGF, ET-1, IL1, IL6, IL8, activin-A, periostin, and COX-2, autocrine stimulatory loops might also play a role in chronic pancreatitis and PDA (Figure 3). Additionally, activated PSC also produce IL15, which reduces lymphocyte apoptosis, again leading to further PSC activation [28].

Accumulating evidence suggests that part of the PSC in fibrotic pancreas are derived from bone marrow [29,30], express several stem/progenitor cell markers such as CD34, Nestin, p75NTR, GFAP, Bcrp1, Aldh, Notch (for review see [31]), and are able to differentiate into other pancreatic cell types [32]. Pan and coworkers have shown that bone marrow-derived progenitor cells contribute to the stellate cell and inflammatory cell population near metaplastic tubular complexes and carcinoma cells [33]. Based on their data, these authors suggest that bone marrow-derived progenitor cells could influence pancreatic cancer growth by modulating tumor microenvironment [33].

## 3. Cell-Cell Interactions between PSC and Carcinoma Cells

Experimental and clinical data indicate that acute and chronic pancreatitis represent potent risk factors for PDA [34,35,36]. Tissue injury (e.g., acute pancreatitis) [37], oncogene activation like Hedgehog/Ras [38,39,40] or Notch [41,42] and TGFα activation [43] induce and accelerate acinar-ductal metaplasia and PanIN development. Notch activity is thought to play a major role in cancer development because TGFα induced acinar-to-duct conversion requires Notch activation [42], and Notch and Kras coactivation cause a rapid acinar-to-duct-like phenotype [41]. Chronic pancreatitis also accelerates Kras-driven PanIN and PDA development [34]. In addition, accumulating evidence indicates that in tissue injury, and pancreatitis activation of PSC, is linked to tumor progression. Erkan and colleagues quantified the extent of activated stromal cells *in situ* by quantifying what they called the “activated stroma index” (ASI) [44]. They stained consecutive tissue sections of cancer patients with antibodies against aSMA or with aniline blue revealing collagen deposition. What they observed was that a high coefficient of aSMA / collagen staining correlated with a poor prognosis and *vice versa*. This indicates that high PSC numbers and a high activation grade of PSC (strong aSMA staining), but not a strong desmoplastic reaction (collagen synthesis), are related to tumor progression.

**Figure 4:**
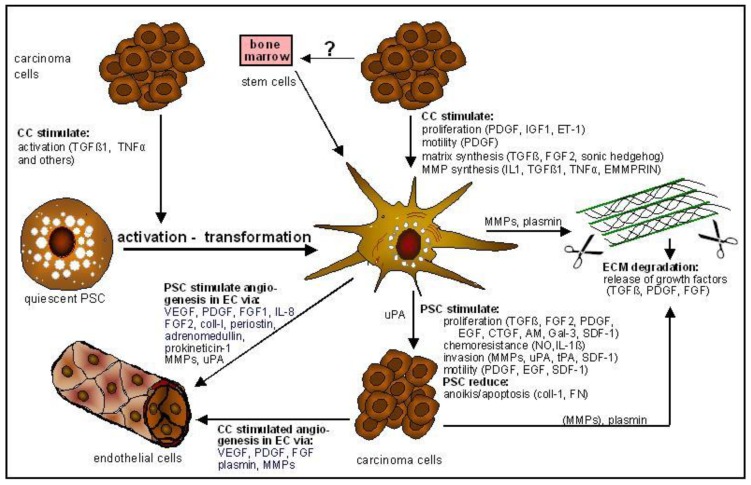
Interaction of PSC with pancreas carcinoma cells. Pancreas carcinoma cells (CC) accelerate transformation of quiescent PSC to the myofibroblast-like phenotype. This cell type is attracted by CC and stimulated to proliferate, produce ECM and growth factors. PSC stimulate angiogenesis, CC proliferation, chemoresistance, invasion, and motility. In addition, PSC reduce anoikis/apoptosis of CC. AM, adrenomedullin; SDF-1, stromal cell‑derived factor-1.

The cell type that gives rise to precursor lesions, termed pancreatic intraepithelial neoplasia (PanIN), is still in debate. Pancreatic duct epithelial cells [45] or centroacinar cells [46,47] have been suggested as cancer-initiating cells, but evidence accumulates that acinar cells represent the bad guys [41,48,49,50]. There are also hints that PSC might participate in the initiation of PDA [51]. Beside the growth factors mentioned above, other factors might also be involved in CC-PSC interaction and contribute to cancer progression (see Figure 4). Firstly, PSC store retinaldehyde esters within lipid droplets, which may be oxidized by aldehyde dehydrogenases to retinoic acid (RA). Upon activation of PSC, the lipid droplets disappear, thereby releasing their contents. RA promotes cell differentiaton—a physiological process needed to maintain tissue homeostasis, e.g., for restricting acinar cell proliferation. Following continuous activation of PSC though, RA stores may be depleted and proliferation of acinar cells may continue once initiated. Secondly, collagen I, which is produced by activated PSC, has been shown to directly weaken E-cadherin mediated cell-cell interactions of tumor cells, and to stimulate cell proliferation by b-catenin-mediated up-regulation of c-myc and cyclin D1 expression [52]. Additionally, collagen I was shown to increase N-cadherin expression and the metastatic potential of CC *in vivo* [53]. In this context, one might speculate that, for example, phagocytosis of necrotic acinar cells [54] or bacteria [55] by PSC may lead to local activation of PSC and collagen production, finally stimulating acinar cell proliferation. In adult mice chronic pancreatitis has been shown to be essential for the induction of ductal adenocarcinomas by K-Ras oncogenes [34], and from humans it is known that both idiopathic and alcoholic pancreatitis are associated with a 15-fold higher risk of developing pancreatic cancer [56]. In summary, the local activation of PSC, simply as a result of local tissue injury, may in turn ‘accidentally’ initiate or promote cell proliferation of acinar cells.

Furthermore, in recent years we have learned from different tumors that the tumor microenvironment plays an active role in tumor progression, invasion, chemoresistance, escape from apoptosis and anoikis and metastasis [57,58,59,60]. At the invasion front, stroma and tumor cells interact and exchange factors (enzymes, growth factors, cytokines) that degrade local ECM and stimulate migration, promote proliferation, angiogenesis and tumor cell survival. Pancreas carcinoma microenvironment contains the angiogenesis stimulating factors VEGF, PDGF, FGF1, FGF2, collagen-I, periostin, adrenomedullin, prokineticin-1, MMPs and uPA, the migration-stimulating factors PDGF, EGF and SDF-1, proliferation stimulating factors such as TGFß, FGF2, PDGF, EGF, CTGF, adrenomedullin [61], Gal-3, and SDF-1, invasion promoting factors such as MMPs, thrombospondin [62], uPA, and tPA, and factors responsible for drug resistance (NO^•^, IL-1ß) and inhibition of anoikis/apoptosis (collagens, fibronectin, laminin). Tumor cells, as well as fibroblasts, myofibroblasts, and in particular activated PSC, are responsible for the production of these factors (see Figure 4).

**Figure 5:**
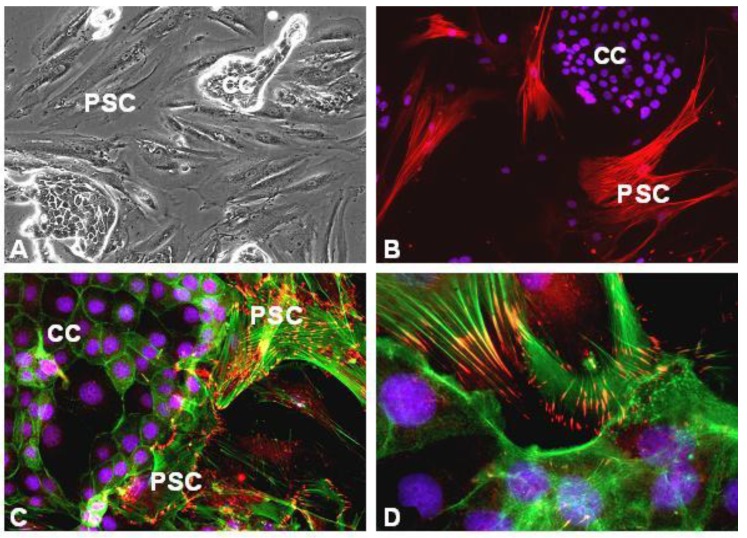
Microphotographs of PSC (isolated from PDA) in co-culture with primary pancreas carcinoma cells (ULA-PaCa). **(a)** phase contrast; **(b)** alpha-smooth muscle actin immunofluorescence (red); **(c)** vinculin, red-yellow, f-actin, green; **(d)** paxillin, red-yellow; f-actin, green. Cell nuclei are stained blue (Hoechst 33258). CC, panceas carcinoma cells (ULA-PaCa); PSC, pancreatic stellate cells.

To study PSC-CC interactions, we performed co-culture experiments of CC and PSC (see Figure 5) or stimulated cultured PSC with CC-supernatants and *vice versa*. The results and the data of others regarding the interaction of PSC with carcinoma cells are summarized in Figure 4. By producing TGFß1 and other fibrogenic mediators, pancreas CC stimulate the transformation of the quiescent fat-storing phenotype of PSC to the highly active myofibroblast-like phenotype. In addition, CC attract PSC (Figure 5c,d) and stimulate motility, proliferation and matrix synthesis of PSC [14,27]. The result of this stimulation is a strong desmoplastic reaction surrounding carcinoma tissue [13,14]. The activated PSC proliferate strongly in response to PDGF, IGF1, and ET-1. Migration of PSC is stimulated by PDGF, matrix synthesis is stimulated primarily by TGFβ1, FGF-2, and sonic hedgehog. In addition, CC induce MMP synthesis via the release of IL1, TGFß1, TNFα, and EMMPRIN. In particular MMP-2, MMP-9, and plasminogen-activator (uPA) are involved in tumor dissemination. As shown by Gress *et al*., MT1-MMP, MT2-MMP, MMP-2, MMP-9 are strongly expressed in pancreas cancer [9]. Recent data from our group shows that PSC significantly contribute to MMP-2 secretion in the desmoplasia of pancreatic cancer *in vivo* and *in vitro* [63]. MMP-2 staining was found primarily in PSC adjacent to cancer cells [14] and secretion of MMP-2 by PSC by far exceeds that of cancer cells [63], although pancreas carcinoma cells express some MMP-2. Furthermore, there is evidence that cancer cells induce uPA expression in stromal cells, which then bind to the urokinase receptor (uPAR) expressed on cancer cells (for review see [64]). After binding, uPA converts plasminogen to plasmin, which then degrades fibrin, collagen IV, fibronectin and laminin. Beside the action of MMPs, this also enables tumor cells to migrate through tumor surrounding ECM. 

In particular, MMPs are suggested to play an important role in cancer progression that is in early metastasis, angiogenesis, and release of growth factors from ECM [65,66]. Early trials with MMP inhibitors though did not result in significant benefits for cancer patients, probably due to their application in late stage cancer, their usage as monotherapy, and missing information about good and bad responders [66]. However, new target-specific inhibitors are being developed [67,68].

Data from our group [14,63] and from the lab of H. Fries [69] have shown that CC express EMMPRIN (Extracellular Matrix Metalloproteinase Inducer) and thereby stimulate the synthesis of MMPs in PSC. EMMPRIN, also named Basigin or CD147, which is a type I transmembrane glycoprotein, has been extensively studied because it is involved in tumor cell migration and invasion [70,71,72,73], apoptosis [74,75], angiogenesis [76,77,78], and chemoresistance [79,80] in a variety of cancers (reviewed by [81]). In pancreatic cancer, serum levels of EMMPRIN are elevated compared to healthy volunteers, though serum levels do not correlate with TNM status [69]. Experiments in nude mice showed that EMMPRIN promotes tumor growth of CC *in vivo* [82].

Most effects of EMMPRIN have been reported to be mediated by the induction of MMPs. For example, positive correlations between the expression of MMPs and EMMPRIN in various cancers can be found in numerous reports [83,84]. Furthermore, co-culture of cancer cells with stromal fibroblasts induces MMPs, which can be blocked by EMMPRIN antibodies [72,85,86]. In addition, there is a positive feedback regulation of EMMPRIN and MMP-dependent generation of soluble EMMPRIN [87]. Finally, the up-regulation of MMP expression in PSC by CC-derived EMMPRIN accelerates tumor growth *in vitro* and *in vivo* [63]. Because of the central role of EMMPRIN in tumor progression, antibody- or siRNA-based therapies have already been tested in mouse models of various cancers with some success [82,88,89]. However, some evidence exists that EMMPRIN does not induce MMP-synthesis in certain cell systems, tumor types, and animal models. In murine melanoma cells, for example, knockdown of EMMPRIN did not reduce the tumor cell-mediated induction of MMP-2, -9, and -14 both *in vitro* and *in vivo* [90], but impaired angiogenesis and metastasis formation [77].

EMMPRIN exists as high and low glykosylated isoform. Their proportion is partly regulated by the interaction of EMMPRIN with caveolin-1 in the Golgi complex [91] and varies between different pancreas CC lines [63,69]. In order to mediate intercellular signals, the transmembrane protein EMMPRIN can be solubilized from the cell membrane by microvesicle shedding [92,93] or by proteolysis of the extracellular part of EMMPRIN by MMPs [87,94]. 

PSC also express varying amounts of EMMPRIN [63,69]. This is of interest, because so far there is no convincing data identifying the receptor for EMMPRIN. The most favored mechanism of action of EMMPRIN is the homophilic interaction of soluble EMMPRIN and membrane-bound EMMPRIN [86,95]. This interaction and the consecutive induction of MMPs can be blocked by unglycosylated recombinant EMMPRIN—the ineffective form of the protein—or by antibodies against EMMPRIN [86]. The main obstacle of this proposed mechanism is the way by which the signal is transduced into the cell. Obviously, EMMPRIN itself has no kinase or phosphatase activity and is not coupled to ion channels or any other known signal transduction molecules. However, following addition of soluble EMMPRIN to cultured cells activation of downstream mediators such as p38-MAPK [96], SAPK/JNK [73], or phospholipase A2 and 5-lipoxygenase [97] have been described.

Another possibility of EMMPRIN signaling might be endocytosis of dimeric or multimeric EMMPRIN (following binding of soluble EMMPRIN) with other components, for example cyclophilin B, which has been shown for the entry of measles virus into epithelial cells [30]. Recently, *in vitro* and *in vivo* experiments have revealed the interaction of EMMPRIN with monocarboxylate transporters, proteins, which confer lactate export from cells in tissues with reduced oxygen supply like solid tumors [82], providing an additional explanation for the tumor promoting effect of EMMPRIN in conjunction with the Warburg hypothesis [98].

Another way EMMPRIN can exert its effects is by the direct binding of MMPs to the tumor cell surface and subsequent local organization of the enzymes in the cell membrane (e.g., a directed localization towards invadopodia), which has been shown for lung carcinoma cell‑derived MMP-1 [99]. This strongly accumulated MMP could again locally activate further MMPs, for example PSC-derived MMP-2 and MMP-9.

Mutual activation and inactivation of MMPs plays an important role in regulation of MMP activity (for review see [100]). Additionally to EMMPRIN (and other factors), these proteases are also tightly regulated by tissue inhibitors of MMPs (TIMPs). In PDA, an imbalance of MMP and TIMP expression compared to healthy controls has been observed [101]. As tumor cells proliferate, the total amount of MMPs (inactive pro-MMPs as well as active MMPs) would concomitantly increase and further activate PSC-derived MMPs. Hereby, a positive feedforward loop on ECM remodeling is generated.

By producing multiple cytokines and growth factors including PDGF, FGF, TGFβ1, CTGF, IL-1β, IL-6, IL-8, SDF-1, Rantes, TNFα, MCP-1, and ET-1 activated PSC also influence proliferation, motility, invasion, and chemoresistance of CC (see Figure 4). In addition, via the production of VEGF, PDGF, FGF1, FGF2, IL-8, coll-I, periostin, adrenomedullin, prokineticin-1, MMPs, and uPA, activated PSC also promote angiogenesis (see Figure 4). CC proliferation is stimulated via the growth factors TGFß, FGF2, and PDGF [13,14], invasion via the production of MMPs, and motility via PDGF and EGF. These data have been confirmed by C. Logsdon’s group which has shown that PSC supernatants increased tumor cell proliferation, migration, invasion, and colony formation [102]. Furthermore, gemcitabine and radiation therapy were less effective in tumor cells treated with PSC supernatant. Although all the responsible mediators have not been identified as yet, activation of MAPK and Akt pathways have been observed after addition of PSC supernatant to cultured tumor cells [102]. Very recently X. Wang’s group identified another soluble factor in PSC supernatant (stromal cell-derived factor-1 = SDF-1) promoting proliferation, migration and invasion of cancer cells [103]. In addition, very interestingly the presence of PSC increased the incidence of tumor formation when limiting numbers of CC were orthotopically injected into nude mice [102]. These data were confirmed by M. Apte’s group using orthotopically transplanted MiaPaCa-2 cells in combination with PSC from different human donors [104]. They identified PDGF as the primarily responsible factor for the tumor promoting effects of PSC (*in vitro*). Interestingly, the group also reported the existence of αSMA-positive human cells in liver nodules together with a higher incidence of distant metastases on transplantation of CC with PSC than without (50% *vs*. 10%), indicating that some PSC might have comigrated with the CC. Our *in vivo* data, showing the induction of subcutaneous tumors in nude mice, also indicate that, in the presence of PSC, tumor progression is accelerated [13,63]. Others have shown that direct contact of tumor cells with PSC activated the Notch signaling pathway and resulted in even stronger stimulation of tumor cell proliferation compared to stimulation by supernatant [105].

In summary, all these observations support the hypothesis that PSC provide a microenvironment which is advantageous for tumor cell growth and survival. Interestingly, several growth factors, such as TGFß, FGFs, PDGF-BB and insulin-like growth factor-I (IGF-1) are sequestered within the ECM, which thus acts as a sponge for these factors [105]. Proteases from CC or MMPs from PSC (which are induced through EMMPRIN from CC) might degrade the ECM and release these bound growth factors.

**Figure 6:**
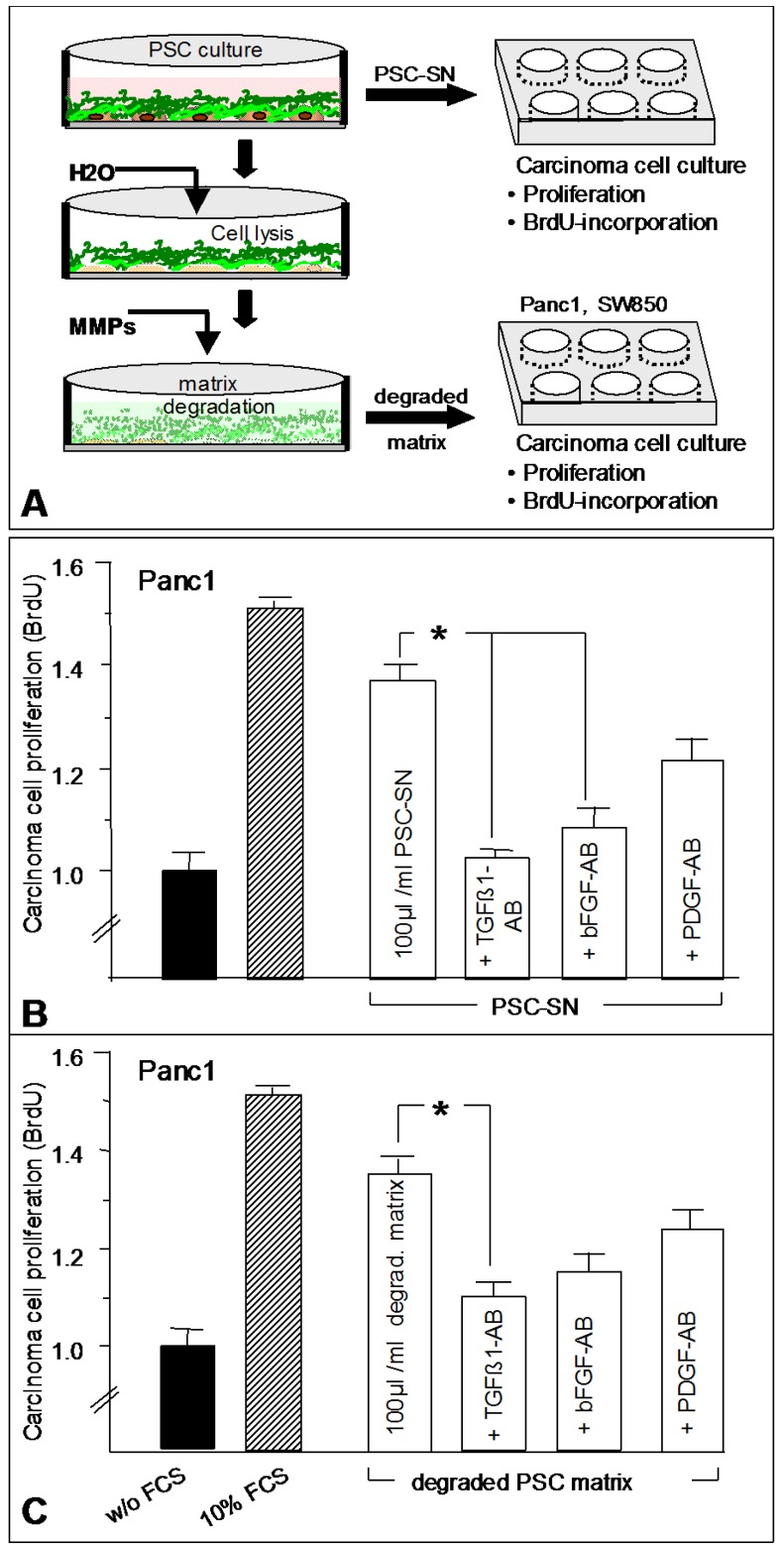
Effect of PSC supernatants (PSC-SN) and degraded extracellular matrix on proliferation (BrdU-incorporation) of cultured Panc1 cells. **(a)** Activated PSC were cultured in 6-well plates until confluency. Then, medium was changed and in the absence of fetal calf serum new medium was conditioned for 3 days. The PSC supernatant was used to stimulate Panc1 cells (see b). The cultured PSC were washed and then lysed using distilled water. After another 3 washing steps the remaining ECM was degraded at 37 °C by addition of 2ml CC supernatant (containing proteases and MMPs). Degraded matrix was preincubated with neutralizing antibodies or directly added to cultured Panc1 cells (see c). **(b)**, PSC supernatant was added to cultured Panc1 and proliferation was quantified by BrdU-incorporation. **(c)** Degraded matrix (with and w/o preincubation with neutralizing antibodies) was added to cultured Panc1 and proliferation was quantified by BrdU-incorporation. * p < 0.05.

As shown in Figure 6, we designed a set of experiments to demonstrate the release of growth factors by degradation of ECM. Activated PSC were cultured in 6-well plates until confluency. Then, the medium was changed and in the absence of fetal calf serum new medium was conditioned for three days. This PSC supernatant was added to cultured pancreas CC (Panc1 and SW850) and proliferation of the CC was quantified by BrdU-incorporation (see Figure 6a and 6b). Cultured PSC were washed and then lysed using distilled water. After a further three washing steps, the remaining ECM was degraded at 37 °C by addition of 2 mL CC supernatant (containing proteases and MMPs). The degraded matrix was then added to cultured CC and proliferation was again measured by BrdU incorporation (Figure 6a and c). In addition, PSC supernatant and degraded PSC-matrix were preincubated for 1 h with neutralizing antibodies against TGFß1, bFGF, and PDGF-AB.

As shown in Figure 6a and 6b, PSC-conditioned medium (PSC-SN) and degraded PSC matrix both stimulate the proliferation of Panc1 cells by almost 40% compared to control. Preincubation with neutralizing antibodies against TGFß1 and bFGF identified both factors as mitogens for CC. Similar results were obtained using SW850 cells instead of Panc1. In summary, these experiments demonstrate that (i) TGFß1 and bFGF are produced by cultured PSC; (ii) both factors are sequestered in the ECM and might be released by matrix degradation; (iii) both factors stimulate proliferation of pancreas carcinoma cell lines.

## 4. Role of PSC in Chemoresistance

As mentioned above, surgical resection of PDA in combination with chemo- or radiotherapy is the only chance for cure. However, only 15–20% of the patients are candidates for surgical intervention. Another problem is the development of chemo- and radioresistance in PDA. The synthetic nucleoside analogue gemcitabine, which is the most commonly used drug for chemotherapy of PDA, is a prodrug, which needs to be transported into the tumor cells followed by activation through various enzymes. Recent data show that, among these proteins, a high expression of equilibrative nucleoside transporter 1, which enables the entry of the compound into the cells, correlates with higher survival in gemcitabine-treated patients [107]. Within cells, gemcitabine is phosphorylated by various enzymes and inhibits ribonucleotide reductase and DNA synthesis [108], but phosphatases, like 5’-nucleotidase, or cytidine deaminase, which irreversibly deactivate the drug, again may limit this process. Additionally, as one might expect, there is a positive correlation between the expression of anti-apoptotic genes of the Bcl-2 family, like Bcl-XL, and resistance of CC against gemcitabine or 5-fluorouracil [109]. Up-regulation of these anti-apoptotic genes is mainly conferred by mutations of nuclear factor kB (NF-κB), variations in signaling pathways upstream of NF-κB, or continuous auto- and paracrine stimulation of NF-κB activity by various growth factors and cytokines. Inhibition of NF-κB increased apoptosis and thereby reduced chemoresistance [110].

Participation of PSC in the above mentioned mechanisms has not been investigated so far, but there are several observations indicating that PSC might play a significant role in both the manifestation and progression of chemoresistance. PSC-conditioned medium, for example, directly protects CC against the cytotoxicity of gemcitabine: Only 9% of BxPC3 cells in the presence, compared to 34% of cells in the absence, of PSC-conditioned medium were TUNEL-positive after 48 hours treatment with 100 µM gemcitabine [102].

Among others, CC express IL-1β, which confers constitutive NF-κB activity and chemoresistance via autocrine stimulation [111]. At this point, PSC come into play: IL-1β leads to the expression of inducible nitric oxide synthase (iNOS) in PSC [112]. PSC do not express IL-1β, but, once expressed, the ‘constitutively’ active iNOS—being independent on enzyme regulators like calcium/calmodulin—releases nitric oxide (NO^•^). The freely diffusible molecule NO^•^ increases, as a paracrine mediator, the expression of IL-1β in CC, resulting in a positive feedback loop. Co-culture experiments using either PSC or PSC-conditioned medium revealed significantly reduced rates of etoposide-induced apoptosis (>50% reduction) in CC compared to CC mono-cultures. This effect was blocked by either an IL-1β receptor antagonist or the iNOS inhibitor aminoguanidine, but enhanced by the NO^•^ donor S-nitroso-N-acetyl-D,L-penicillamine (SNAP). Additionally, histology revealed both the expression of IL-1β and iNOS in human pancreatic adenocarcinoma samples [112]. In this context, hypermethylation of caspase-3, -7, -8, and -9 genes, resulting in reduced expression of these effector enzymes of apoptotic signaling, seems to be responsible for the increased resistance to apoptosis [113].

Another research group has shown that culture of CC on ECM protein-coated dishes (including fibronectin, laminin, collagen type I and IV) directly influences CC proliferation and increases the resistance of CC against the cytotoxicity of 5-fluorouracil, cisplatin, and doxorubicin [114]. This suggests that ECM proteins, abundantly expressed by PSC, might directly promote resistance against anticancer drugs. Interestingly though, none of these ECM proteins changed gemcitabine-mediated cytotoxicity (75–400 nM; 72 hours) in any CC line investigated (Capan-1, Panc-1, MiaPaCa-2) [114], indicating that the increased resistance against anticancer drugs mediated by ECM proteins alone does not adequately reflect the situation *in vivo*.

Finally, multicellular layer experiments, used as *in vitro* models for solid tumors, suggest a limited penetration of anticancer drugs through tumor tissues [115]. In case of PDA, a possible candidate gene directly involved in such an increased chemoresistance is the glycoprotein decorin, which is highly expressed on both the mRNA and protein level in activated PSC. It leads to decreased PCC proliferation, but increased gemcitabine resistance, probably due to direct binding of growth factors (affecting proliferation) [116] and small molecules like gemcitabine via it’s leucine-rich domains. The net effect of decorin on proliferation (inhibition) and chemoresistance (increase) *in vitro* is to slow down CC growth [117].

A recent study reported major progress with respect to reducing chemotherapy resistance in PDA. Inhibition of hedgehog signaling (involved in tumor-stroma interaction as mentioned above) in a murine model of PDA, reduced stromal expansion, thereby increasing intratumoral vascular density and intratumoral concentration of gemcitabine, leading to transient stabilization of disease [118].

In summary, accumulating data indicate that PSC participate in the development of chemoresistance in PDA. Accordingly, research focusing on the improvement of anticancer drug efficiency should not exclusively study CC, but always consider the influence of stromal cells.

## 5. Conclusions

In recent years, it has been established that cancer growth and spread are strongly influenced by the microenvironment. However, presently the molecular signals involved in the tumor-host cross-talk have only partly been identified. Hopefully in the near future, new therapeutic options might be developed, which directly interfere with the tumor-host cross-talk. The most promising cellular target for anti-stromal treatment could be the matrix and growth factor producing PSCs and endothelial cells playing a central role in angiogenesis. In chronic pancreatitis, cancer initiation and progression might be inhibited, also through inactivation of PSC, causing a reduction of matrix remodeling. Recently, it has been shown that forced expression of peroxisome proliferator‑activated receptor g (PPARγ), or CCAAT/enhancer binding protein a (C/EBP-α), induced a phenotypic switch from activated to quiescent PSCs *in vitro*, which was dependent on the expression of albumin [119]. Effects on MMP expression and net effects on tumor progression *in vivo,* however, await further experiments.
